# Incorporating Birch Bark Suberinic Acid Residue Powder into Structural Particleboards: Exploring Fractional Influence on Material Properties in Circular Economy Framework

**DOI:** 10.3390/ma17235750

**Published:** 2024-11-24

**Authors:** Anita Wronka, Grzegorz Kowaluk

**Affiliations:** Institute of Wood Sciences and Furniture, Warsaw University of Life Sciences—SGGW, Nowoursynowska St. 159, 02-776 Warsaw, Poland

**Keywords:** OSB, structural particleboard, circular economy, recycling, SAR

## Abstract

This study investigates the effects of suberic acid residue (SAR) additions on structural single-layer particleboard (like the P5 type, according to EN 312) properties, specifically the water absorption (WA), thickness swelling (TS), modulus of rupture (MOR), modulus of elasticity (MOE), screw withdrawal resistance (SWR), and internal bond (IB) strength. The results indicate that finer SAR fractions (1/0.25 and 2/1) reduce the WA after 2 h of soaking, while larger fractions increase the WA after 24 h, with only the smallest fraction meeting the TS standards. The MOR values (18.5–19.6 N mm^−2^) and MOE (3627–3811 N mm^−2^) remain largely unaffected by SAR additions, while the SWR shows minimal variation across various SAR fractions (203–209 N mm^−1^). The IB strength improves with SAR additions, peaking at 2.10 N mm^−2^ for the 5/2 fraction, though slightly decreasing with the largest fraction (8/5). A density analysis reveals an increased surface density with finer SAR fractions, benefiting the surface strength but reducing the core uniformity with larger fractions. These findings suggest that SAR-enhanced particleboards could be valuable in applications requiring moisture resistance, such as bathrooms, kitchens, and exterior cladding. Further research should explore optimizing the SAR concentration, combining it with hydrophobic agents, and examining its long-term stability under varying environmental conditions to enhance its structural performance for sustainable building applications.

## 1. Introduction

Type P5 wood-based boards are construction materials designed for environments with elevated humidity, due to their high moisture resistance. Classified under European standards EN 312 [1] and EN 300 [2], these boards include both particleboard and oriented strand board (OSB) that offer enhanced strength and water resistance. This makes them suitable for applications in areas such as kitchens and bathrooms, where exposure to moisture is common. OSB is a widely used composite material used mainly in construction and furniture manufacturing; in flooring systems, it acts as a flexural or shear element, offering a solid and sturdy foundation, as confirmed in the research of Schwab et al. [3]. Iuorio et al., in their research, also give examples of why it is prized for its strength, dimensional stability, and versatility [4]. Because of its strength, adaptability, and affordability, OSB is frequently used in new residential construction in North America, especially for wall sheathing, roof panels, and floor underlayment, as confirmed by Baffa et al. [5], Gjinolli et al. [6], and Boardman et al. [7]. OSB panels are made from thin, compressed wood strands arranged in alternating layers, which endows them with outstanding mechanical properties, including high resistance to bending and tensile forces. Many kinds of wood, including hardwoods like beech and poplar and softwoods like pine, are frequently the source of these strands. At greater densities, panels with 50/50% of beech–poplar/beech–pine/poplar–pine exhibited better mechanical qualities, especially regarding the internal bond values and bending strength, which is in accordance with the research of scientists Akrami et al. [8,9], Yano et al. [10], and Via et al. [11], who have claimed that, to minimize the environmental effect and cut costs, using lignocellulosic materials in OSB production could be a sustainable approach to repurposing agricultural waste.

Alternative species, including birch, maple, and other underused woods, are being investigated due to conventional wood supplies’, like aspen and spruce, depletion. However, the performance of OSB panels can be significantly impacted by these substitutes, necessitating a great deal of study to improve production procedures and panel structure, as confirmed by the research of Wan et al. [12] and Pipíška et al. [13]. Domingos et al. [14] showed that by incorporating byproducts like sawdust and eucalyptus bark—materials that would otherwise become industrial waste—OSB production reduces the demand for virgin wood and advances environmental sustainability. Utilizing these waste materials for OSBs minimizes the environmental impact and supports resource efficiency in building materials, aligning closely with circular economy principles. Other raw materials that have been utilized to fabricate OSBs include tali and moso bamboo. The research of Iswanto et al. [15] and Suzuki et al. [16] has shown that bamboo can enhance OSBs’ mechanical qualities, including its modulus of elasticity (MOE) and modulus of rupture (MOR). Aspen wood and citrus branches are examples of lignocellulosic flakes that have shown a higher MOR and MOE, indicating improved mechanical strength, as evidenced by the studies of Tichi et al. [17]. The potential of poplar, willow, and birch to take the place of conventional softwoods in OSB manufacturing has been assessed. Birch OSB demonstrates better elastic qualities, and these species satisfy the minimal criteria for OSB/2 characteristics, as was confirmed in the research by Dumitrascu et al. [18]. Da Silva et al. [19] used sugarcane bagasse as an OSB raw material to repurpose this waste product in a useful way. Sweet chestnut wood has demonstrated intriguing promise for producing load-bearing OSBs with increased fungal resistance, making them appropriate for usage in settings where fungal degradation may occur, which was examined by Thévenon et al. [20]. It has been demonstrated in the research conducted by Pędzik et al., that using walnut wood residues, a waste or by-product of the wood-processing industry, can effectively improve particleboard’s mechanical qualities, suggesting the possibility of using wood residues as an alternate raw material in the production of composites [21]. With similar MOR and MOE values as those made from spruce, OSBs made from European larch, poplar, willow, and alder have demonstrated potential as natural alternatives to traditional wood-based OSBs, suggesting their suitability for producing OSBs without combining species, which Pipíška et al. confirmed [13]. Baskara et al. investigated the potential use of African, Mangium, and Sengon wood species in OSB production, which was shown to improve the mechanical and physical characteristics of OSBs formed from these wood strands with an increased resin content [22]. Lee et al. [23] claimed that materials like straw, stalks, bagasse, seeds/fruit, leaves, grass, and palms are abundant post-harvest and can significantly reduce production costs while adhering to circular economy principles.

Using alternative wood species in OSB production offers potential cost savings and environmental benefits; however, meeting the technical standards—such as those for thickness swelling—can be challenging, potentially limiting the boards’ suitability for specific applications. Further research and development are essential, particularly for refining the “transferable durability” approach, which aims to enhance the durability of perishable wood species by using natural extractives from durable wood species. This approach faces obstacles, including optimizing the extraction methods and developing effective preservative formulations, as confirmed by Pędzik et al. [21], Manhiça et al. [24] and Mishra et al. [25].

In addition to the raw material factors, the quality of OSBs is also affected by production factors, such as the press factor and line speed; mechanical qualities like the MOE and MOR are improved by raising the press factor and lowering the line speed, as evidenced by the research of Ciobanu et al. [26]. Carneiro et al. [27] showed that other crucial elements are the temperature and pressure; while greater temperatures and pressures during compaction enhance the flexural qualities and hardness, they can also increase thickness swelling and water absorption. The strand length and density, and the MOE, MOR, and shear strength are improved by longer strand lengths and greater densities, which was confirmed by the research of Chen et al. [28]. Regarding the strand geometry, the MOE and MOR are influenced by the strands’ slenderness ratio (length/thickness), with thinner strands enhancing these characteristics; Iwakiri et al. drew these conclusions based on their research [29]. The wood’s density also influences the ultimate performance; woods with a higher density, like Cambará, have better physical qualities, while woods with a lower density, like Pinus, have better mechanical attributes, which was observed by Salles et al. [30]. The physical and mechanical properties of OSBs are significantly influenced by their resin content. Increased resin levels, determined by the initial resin concentration, enhance critical characteristics, such as the internal bond strength and MOR. The relationship between the resin content and board performance is further evidenced by the correlation between the mechanical properties and thickness swelling, indicating that the final resin content plays a critical role in determining the structural integrity and durability of OSBs, as shown by Taheri et al. [31].

The layered structure of OSBs makes them a cost-effective and often more sustainable alternative to traditional plywood, while providing the durability and strength required for a wide range of applications. As the construction industry increasingly prioritizes sustainability, OSB manufacturers are exploring innovative ways to enhance production efficiency and reduce the environmental impact. One promising advancement involves using suberin-rich waste fractions—the byproducts of extracting suberic acid from birch bark—as additives in OSB production. These fractions, derived from chemical extraction processes, can improve OSBs’ mechanical and thermal properties, decrease reliance on virgin wood, and reduce the emissions associated with building materials. Additionally, incorporating post-extraction birch bark waste can lower formaldehyde emissions compared to conventional sealing methods used in traditional composites, further supporting healthier and more sustainable building practices, as claimed by Jeżo et al. [32].

The practical applications of P5 boards modified with an SAR additive may be similar to those of the same boards without modification. However, one of its main advantages may be its lower formaldehyde emissions than that of its counterpart without SAR. SAR can absorb formaldehyde, as mentioned by Maksymiuk et al. [33]. Lower formaldehyde emissions contribute to improved indoor air quality, enhancing the overall hygiene and healthiness of spaces. Reducing harmful emissions helps create a safer and more comfortable environment for the occupants, promoting better well-being in both residential and commercial settings, as confirmed by the research by Jung et al. [34]. Due to its consistency, stability, and affordability, particle board is widely used in furniture production, including for cabinets, tabletops, and sliding doors; numerous examples of these were mentioned by Rashid et al. [35].

The most important publications and their conclusions are summarized in Table 1.

This article aims to analyze the impact of various fractions of post-extraction birch bark waste on the properties of structural particleboard, focusing on its potential applications within a circular economy—a growing priority in modern construction.

## 2. Materials and Methods

### 2.1. Materials, Preparation, and Characterization

A single-layer structural particleboard with a nominal density of 800 kg m^−3^ was produced, featuring dimensions of 320 mm × 320 mm and a thickness of 12 mm. The primary raw material used to produce the structural particleboard was *Pinus sylvestris* L. The particle fractions used in the structural particleboard ranged from below 8 mm to 2 mm. Different size fractions of SAR kiln-dried at 70 °C over 3 days were produced, selected through sieving, and then added to the structural particleboard. The fraction that passed through an 8 mm sieve but was retained by a 5 mm sieve (8/5) was used for this study. Other fractions were similarly obtained as follows: 5/2, 2/1, and 1/0.25 mm. The SAR content (*w*/*w*) in each variant was 10%. The SAR was sourced from the Latvian State Institute of Wood Chemistry in Riga, Latvia. It was produced from residual byproducts generated during the extraction of suberinic acid (SA), using the procedure detailed by Makars et al. [36]. The SAR water absorption method has been described by [37]. The resination of the boards was 15%, and a commercially available melamine–urea–formaldehyde (MUF) (Silekol Sp. z o.o., Kędzierzyn-Koźle, Poland) resin was used with a melamine content of 5.2%, a molar ratio of 0.89, and a solid content of 66.5%. A paraffin emulsion was added; its content was 1% and the ammonium nitrate (hardener) content was 5%, both calculated regarding the dry resin content.

Following the pressing process, the particleboards were sanded to ensure uniform thickness. A control particleboard was produced using the same method but without SAR inclusion. Before conducting any tests, all the panels were conditioned for seven days at 20 °C and 65% relative humidity. The particleboards were pressed using an AKE hot press (Mariannelund, Sweden) under conditions resembling those of industrial practices: a temperature of 200 °C, a peak specific pressure of 2.5 MPa, and a pressing duration of 20 s mm^−1^ of the panel’s nominal thickness. This study evaluated the mechanical and physical properties of the particleboards, including the MOR and MOE, following the relevant European standards where applicable [38]; the internal bond (IB) strength was measured in accordance with the EN 319 standard [39]. The screw withdrawal resistance (SWR) [40], water absorption (WA), and thickness swelling (TS) were measured after 2 and 24 h of immersion in water [41]. All the mechanical properties were evaluated using a computer-controlled universal testing machine (Research and Development Centre for Wood-Based Panels Sp. z o.o., Czarna Woda, Poland). Each mechanical and physical test involved at least eight samples per panel type. For the density profile (DP) measurements, specimens measuring 50 mm × 50 mm were assessed with a Grecon DA-X device (Fagus-GreCon Greten GmbH & Co. KG, Alfeld/Hannover, Germany) using direct X-ray densitometry, scanning the panel thickness in 0.02 mm increments. A representative density profile was selected after analyzing three samples of each variant for further examination. Where applicable, the results were compared to the European standards [1].

### 2.2. Statistical Analyses

The statistical analyses, including the analysis of variance (ANOVA) and *t*-tests (α = 0.05), were performed using IBM SPSS Statistics software (version 20, IBM, Armonk, NY, USA) to identify the significant differences between factors and levels. Comparative evaluations of the mean values for the MOR, MOE, IB, SWR, TS, and WA were conducted, and homogeneous and non-homogeneous groupings were established. Where applicable, the mean values along with the standard deviations were represented on graphs as error bars to illustrate the variability.

## 3. Results

### 3.1. Water Absorption, Thickness Swelling, and SAR Fractions

**Figure 1:**
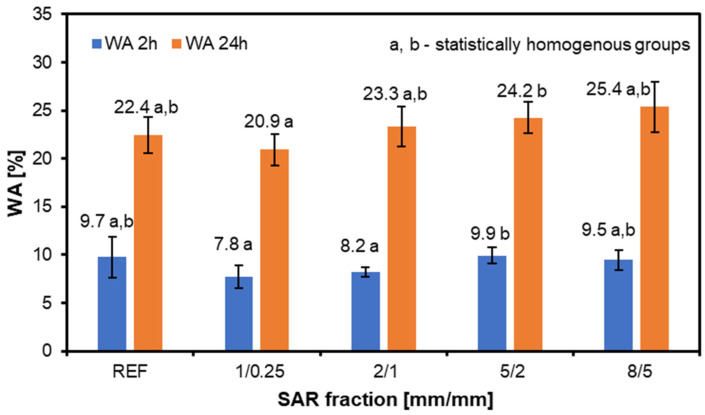
The water absorption of the structural particleboard produced with the addition of various fractions of SAR.

Figure 1 presents the water absorption graphs for the structural particleboard panels produced by adding various SAR fractions. Based on the results obtained after 2 h of soaking the samples, a reduction in the water absorption is observed with the addition of SAR compared to the reference sample—this effect is most noticeable for the smaller SAR fractions, 1/0.25 and 2/1. In the other variants, the results are comparable to the reference samples. Analyzing the dimensional changes after 24 h of soaking, it can be noted that the larger the fraction added to the board, the higher the WA.

SAR increases the water absorption when added to materials like particleboards for several reasons relating to SAR’s chemical makeup and characteristics. SAR comprises hydrophilic substances, such as cellulose, lignin, esters, and suberin monomers. Since these substances have a strong affinity for water, adding SAR to materials increases their ability to absorb water. The addition of SAR reduces the composite material’s hydrophobicity. Because of this decrease in hydrophobicity, the material may absorb and permeate more water [33]. The formation of water molecular bridges (WaMBs) and the synergistic effect of polar and apolar domains are two complementary processes via which the structure of SAR promotes water retention and transport. Like humic acids, SAR’s polar groups give water molecules main sorption sites where they may form WaMBs when humidity levels rise. These bridges raise the water content by facilitating water flow through a material. At the same time, SAR’s polar and apolar domains work in concert to improve water binding and stability, giving it more flexibility and retention in situations with varying humidity levels. SAR is a valuable chemical for enhancing the water management qualities of materials exposed to different ambient moisture levels because of its dual action [42]. The cell walls of some plant tissues, such as the root endodermis, contain suberinic acid residues, which help establish a protective barrier that regulates ion and water transport and increases water absorption [43,44]; based on this information, it can be concluded that suberic acid residues remaining in waste may act similarly. Maksymiuk et al. [33] added a dusty fraction to a three-layer particleboard; their study confirmed that a 5% SAR share reduced the WA of the samples tested, which is in line with the research we conducted. Here, it was also confirmed that the smallest fraction contributed to the lowest WA.

**Figure 2:**
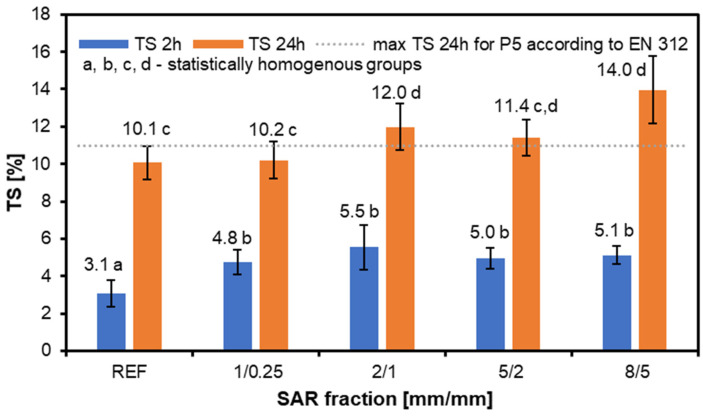
The thickness swelling of the structural particleboard produced with the addition of various fractions of SAR.

Analyzing the TS of the tested structural particleboard (Figure 2) panels revealed that, after two hours, the TS was more pronounced for the 1/0.25 and 2/1 variants compared to the water absorption (WA). However, each variant’s TS values exceeded those of the reference samples. After 24 h, the trend became similar to that observed for the WA, increasing with the size of the SAR addition. The results indicate that only the variant with the smallest SAR fraction meets the standard for TS. This research confirms that adding more than 10% SAR negatively impacts the TS performance of particleboard. However, it is essential to consider that factors, such as the glue type, adhesive application, and hydrophobic agents, can also significantly influence TS values [45,46]. To improve the properties of structural particleboard, especially in terms of the water absorption and thickness swelling tests, it is possible to use exotic wood, which improves the results of the above tests. Nevertheless, this is a good solution for countries with easy and cheap access to such raw materials [47].

**Figure 3:**
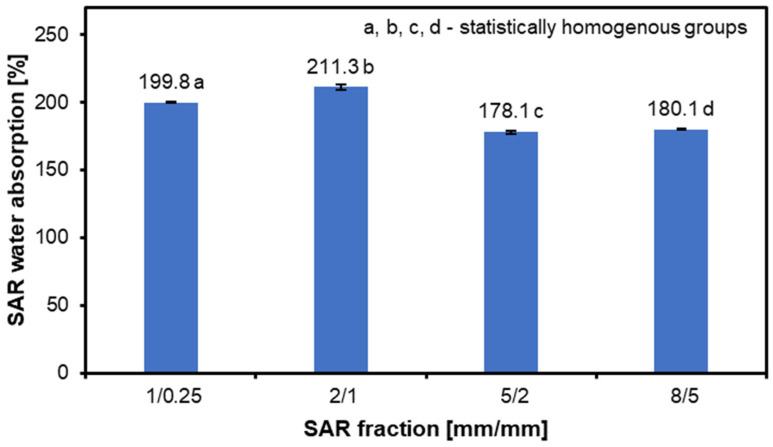
SAR water absorption test for different fractions.

Figure 3 shows the water absorption results for each SAR fraction after 10 min of soaking in water. The results show that the fraction 2/1 has the highest water absorption of 211.3%, while fractions 1/0.25 and 2/1 have comparable absorption values of 199.8% and 211.3%, respectively. The 5/2 fraction achieves a value of 178.1%, and the 8/5 fraction has a value of 180.1%. The size of the dust or particles utilized, the content of the substance, and the duration of immersion all affect how much water is absorbed [48,49,50]. The values obtained are similar to those for hazelnut shell meal, but when subjected to carbonization result in dust [37]. Therefore, it can be concluded that the obtained results follow those of the literature data, as the origin of the tested raw material and the size of the fraction translate into the obtained results. The degree of fineness affects the availability of free spaces, which water can fill, but only to a certain extent.

### 3.2. Determination of MOR and MOE in Bending

**Figure 4:**
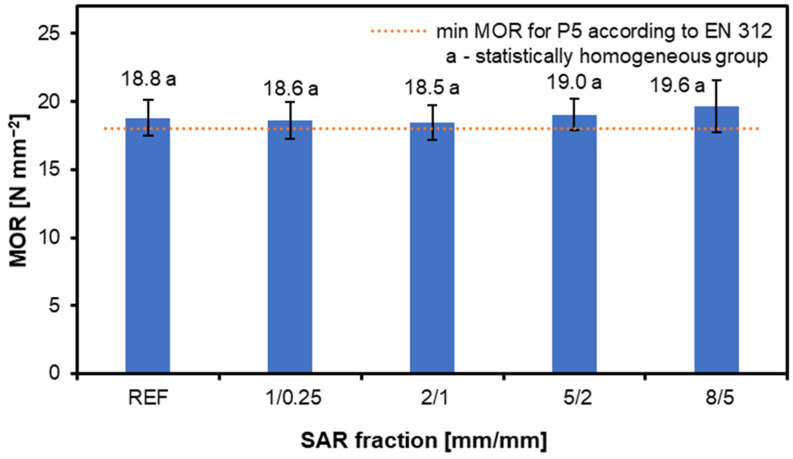
Influence of various amounts of SAR addition on MOR of produced structural particleboard.

Figure 4 displays the MOR values for the structural particleboard panels with varying SAR fraction sizes. Each bar indicates a distinct SAR particle size fraction, from the smallest (1/0.25) to the biggest (8/5). All of the manufactured variants meet the standard’s requirements, and the addition of SAR does not significantly affect the MOR values. The reference MOR value is 18.8 N mm^−2^, with the lowest MOR observed for the 2/1 variant, at 18.5 N mm^−2^, and the highest for the 8/5 variant, at 19.6 N mm^−2^. The addition of SAR can be considered neutral, which is ultimately a positive conclusion, because it does not worsen the MOR of the structural particleboard. The literature suggests that adding up to 10% of SAR can positively influence the MOR, whereas higher concentrations tend to reduce this value [33]. Studies using various materials, such as rice husks and maize cobs, have shown that larger particle sizes typically increase the MOR [51,52,53].

It is worth noting that values such as the MOR are also affected by the thickness of the particleboard; the smaller the thickness, the lower the MOR value is, and the particleboards are more susceptible to deflection. As a result, the manufactured boards with 12 mm MOR values are not too high, which is in line with the results of other researchers [35]. Other studies have reported that the MOR of particle boards containing 50% black spruce bark is lower than that of control boards [54], so SAR is a much better solution because it does not negatively affect the strength parameters.

**Figure 5:**
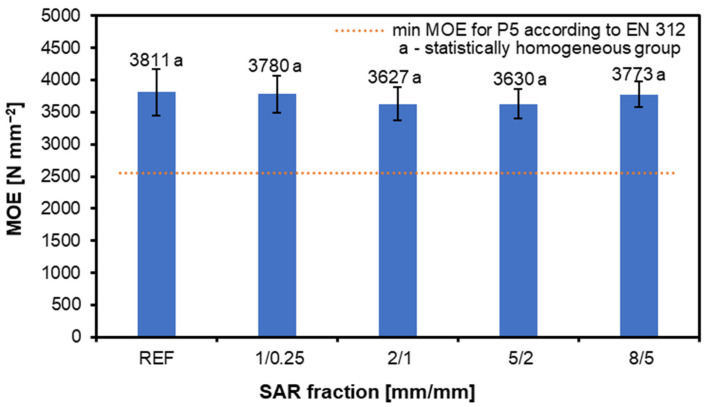
Influence of various amounts of SAR additions on MOE of produced structural particleboard.

Figure 5 shows the MOE results for the tested samples, with the following values recorded: REF—3811 N mm^−2^, 1/0.25—3780 N mm^−2^, 2/1—3627 N mm^−2^, 5/2—3630 N mm^−2^, and 8/5—3773 N mm^−2^. Similar to the MOR results, no significant effect of the SAR additive on the MOE of the samples is observed. This is a positive outcome, as all the variants meet the standard requirements. The SAR additive accounts for 10% of the composition of the boards, so its influence may not be so evident for the example of the MOE results, as many factors can affect the values obtained; these include the length of the particles used [55], the density of the boards [56], sealing [57]. A higher fine content can negatively impact the MOE and MOR in the main direction, but it does not affect the MOE in the core layer [28,58].

The MOE was little affected when hemp shives (10% and 25%) were substituted for wood in the particleboards, suggesting that hemp shives may be used without materially reducing the boards’ elasticity [59]. It can be concluded that similar results were obtained, as the impact of SAR was insignificant and the boards still met the standard’s requirements. Canola straws’ potential as a substitute raw material was demonstrated by the notable improvement in the particleboards’ MOE [60], which confirms that the origin of the alternative raw material for particleboard/OSB production can also favorably influence the performance of, for example, the MOE.

### 3.3. Screw Withdrawal Resistance

**Figure 6:**
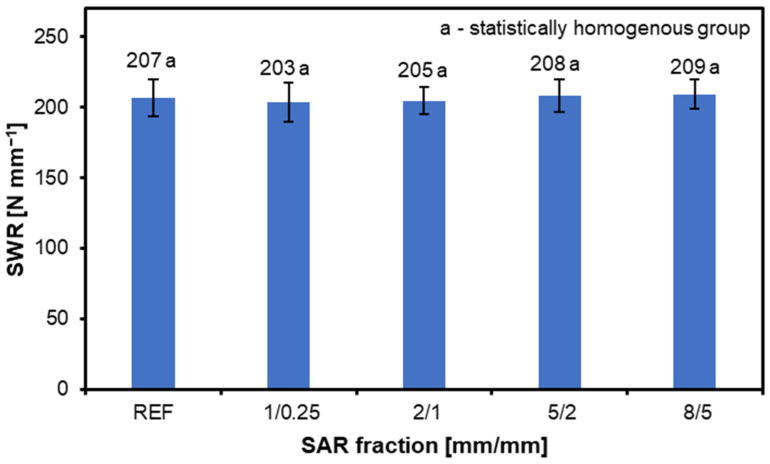
The screw withdrawal resistance of the structural particleboard produced with the various amounts of SAR additions.

Figure 6 shows the results of the screw withdrawal resistance tests. The results for each variant are as follows: REF—207 N mm^−1^, 1/0.25—203 N mm^−1^, 2/1—205 N mm^−1^, 5/2—208, and 8/5—209 N mm^−1^. The discrepancies between the variants are negligible, so the size of the SAR fraction does not significantly affect the SWR results. The rationale for this phenomenon may be that the amount of SAR is so small that it does not affect the SWR results. Still, in contrast to traditional materials, panels reinforced with natural fibers, such as coir and pejibaye, showed reduced pullout resistance [61]. Conversely, incorporating willow and energy poplar enhanced the SWR of particleboard, as the proportion of these alternative raw materials increased [62]. Tests were also conducted using three different wood particle fractions sealed with pure SAR. That study concluded that the SWR also rose accordingly as the particle fraction increased [63]. Due to the use of two different binders, a reliable comparison with this case is challenging. SAR would need to be added as an admixture to the adhesive mass, similar to the MUF used in the studies that were conducted here. The SWR was marginally increased when using insect rearing waste and rice husks in particleboard at ratios of 10–30. Nevertheless, the SWR dramatically dropped when the residue ratio rose beyond 30% [64]. Thus, depending on the origin and structure of the alternative raw materials, the SWR results may decrease, remain unchanged, or increase.

### 3.4. Internal Bond

**Figure 7:**
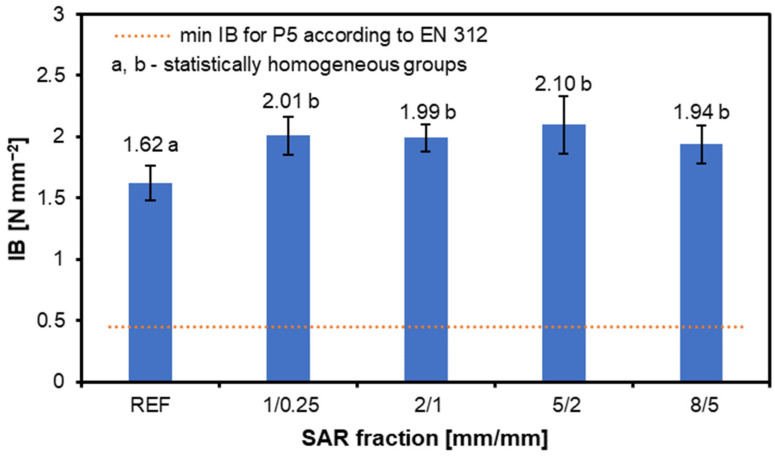
The internal bond of the structural particleboard produced with the various amounts of SAR additions.

Figure 7 presents the IB results for the tested structural particleboard, with the following values recorded: REF—1.62 N mm^−2^, 1/0.25—2.01 N mm^−2^, 2/1—1.99 N mm^−2^, 5/2—2.10 N mm^−2^, and 8/5—1.94 N mm^−2^. Adding SAR generally improves the IB results, as all the modified variants show higher values than the reference. However, the IB parameters decrease when the SAR material was derived from the 8/5 sieve range. The literature indicates that the composition of the core layer significantly affects the IB of the tested boards. Increasing the fine content in the core layer from 10% to 50% enhances IB strength, with the highest values observed at a 30% fine content [58,65].

**Figure 8:**
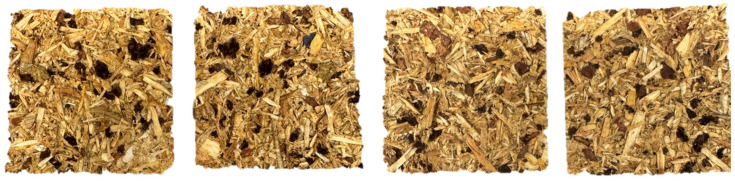
View of samples with 8/5 SAR fraction after IB testing.

Figure 8 shows samples of the 8/5 variant after IB testing. As expected, the specimens cracked in the middle during testing, which is consistent with the density profile. Such a profile has a lower density in the middle layer and, thus, is weaker than the type of forces used in testing [66]. It is noteworthy that the SAR particles cracked symmetrically during the IB test, so its structure is more brittle than wood. According to the IB chart, it can be seen that the IB is weakest at larger SAR fractions—at the same time, this means that there are larger areas of “fragile zones” that contribute to lower IB scores compared to the other samples with SAR added.

For the production of the particleboard, sunflower stalk particles sized 1–3 mm were used in both an unmodified form and after treatment with acetic anhydride and acetone, following a specific procedure. Similar to SAR, this treatment results in a chemically modified raw material. However, this modification led to a lower internal bond (IB) strength compared to the reference results [67].

### 3.5. Density Profiles

**Figure 9:**
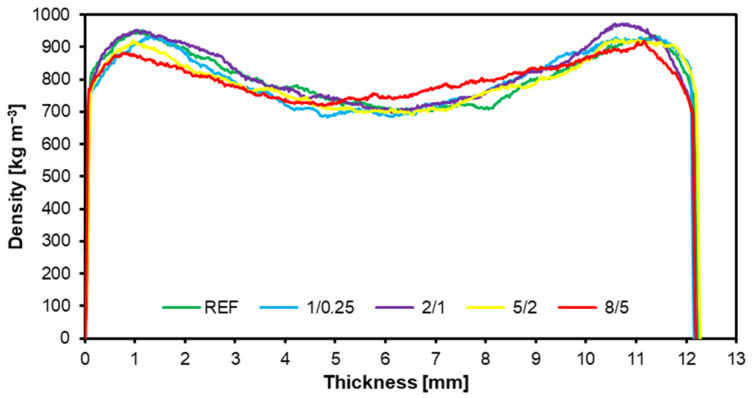
Density profiles of tested structural particleboard.

The density profile graph (Figure 9) shows the density distribution across the thickness of the structural particleboard samples with various formulations. As is typical for structural particleboard, each variant shows distinct density peaks near the board’s surfaces (at the beginning and end of the thickness axis). While the core layer is less dense, the surface layers are denser to increase strength and abrasion resistance. The reference sample, without an SAR addition, is characterized by the classic structural particleboard density profile, with a significantly lower core density and surface peaks. The variant with the finest SAR fraction, which was passed through a 1 mm sieve and was retained by a 0.25 mm sieve, exhibits a density profile similar to that of the reference sample, with a slight increase in its surface density. This suggests that fine SAR fractions can subtly enhance the surface properties without significantly affecting the core. The 2/1 variant has a slightly higher SAR fraction. It shows the highest density values at the surface and a more uniform density in the core compared to the reference sample. This suggests that the larger SAR particles may contribute to better slab strength in the surface and core layers. The 5/2 (yellow) and 8/5 (red) variants have the largest SAR fractions. Higher density peaks are observed on their surfaces, especially for the 8/5 variant, but the core is less uniform. Large SAR fractions can increase the surface density but lead to a less uniform structure in the core, affecting mechanical properties such as flexural strength and dimensional stability.

As the size of the SAR fraction increases (from 1/0.25 to 8/5), the density of the surface layers increases, which can be beneficial for the strength properties. However, with very large fractions (8/5), there is a decrease in the uniformity of the core, which can affect the overall stability of structural particleboard. The method of dosing SAR into structural particleboard within granules of different fractions, and thus excluding the dust fraction, significantly improves the quality of board bonding, as evidenced by the much smaller discrepancies between the top and core layers of the structural particleboard, compared to the particleboard into which powdered SAR was added [33].

## 4. Conclusions

Based on the findings of this study, the incorporation of SAR into structural particleboard demonstrates varying effects on the key properties, depending on the particle size and concentration. The following conclusions summarize the impacts of an SAR addition on the mechanical properties, water resistance, and internal bonding strength of particleboard.

Adding SAR to structural particleboard influences the water absorption, with smaller SAR fractions (1/0.25 and 2/1) reducing the WA after 2 h of immersion. However, higher SAR fractions increase the WA after 24 h, indicating that finer SAR particles enhance the hydrophobic properties. Only the smallest SAR fraction meets the TS standards, suggesting that SAR additions over 10% may negatively impact the TS performance.

SAR additions have a minimal impact on the MOR, with values ranging between 18.5 and 19.6 N mm^−2^, within the standard requirements. The MOE also shows little variation across the SAR fractions, with values from 3627 to 3811 N mm^−2^, indicating SAR’s neutral effect on the bending strength.

The SWR values across the SAR variants (203–209 N mm^−1^) show no significant change, suggesting that SAR has a limited influence on the SWR when used in small amounts.

The IB strength improves with an SAR addition, reaching a peak of 2.1 N mm^−2^ for the 5/2 fraction. However, the IB slightly decreases for the largest SAR fraction (8/5).

Finer SAR fractions increase the surface density without affecting the core density, thus enhancing the surface properties. In contrast, larger fractions (8/5) compromise core uniformity, potentially affecting panel stability.

The controlled addition of SAR can enhance specific properties of structural particleboards, with the effect varying based on the particle size and target application. Finer SAR fractions improve surface strength and water resistance, while larger fractions may limit internal uniformity.

## Figures and Tables

**Table 1:** Overview of studies and key findings.

Group	Key Findings	Source
Material types (OSB, particleboard, and P5)	Due to their strength, moisture resistance, and affordability, OSB and particleboard are used for construction and furniture manufacturing. P5 boards are ideal for high-humidity environments, classified under EN 312 and EN 300 standards.	[1,2,3,4,5]
Wood species, mechanical properties, and sustainability	Using mixed wood species, like beech–poplar and poplar–pine, enhances OSBs’ mechanical properties, such as their internal bond and bending strength. Alternative species, like birch, maple, and bamboo, can also be used, but their performance may vary. Incorporating waste materials, like sawdust, eucalyptus bark, and sugarcane bagasse, reduces the demand for virgin wood and supports environmental sustainability. Additionally, formaldehyde emissions are reduced with SAR-modified boards, contributing to a healthier indoor air quality.	[6,7,8,9,10,11,12,13,14,15,16,17,18]
Production factors and environmental impact	Factors, such as the resin content, strand length, and density, significantly affect OSBs’ mechanical properties. Additionally, using agricultural byproducts like straw and fruit seeds can lower production costs and the environmental impact while promoting sustainability.	[19,20,21,22]

## Data Availability

https://doi.org/10.18150/XBCDSS (created and accessed on 2 November 2024).
